# Health system-related needs for healthy nutritional behaviors in adolescent girls with polycystic ovary syndrome (PCOS): a qualitative study in Iran

**DOI:** 10.1186/s12913-022-08334-2

**Published:** 2022-08-05

**Authors:** Leila Hajivandi, Mahnaz Noroozi, Firoozeh Mostafavi, Maryam Ekramzadeh

**Affiliations:** 1 Department of Nursing and Midwifery, Kazerun Medical Sciences Branch, Islamic Azad University, Kazerun, Iran; 2 Department of Midwifery and Reproductive Health, School of Nursing and Midwifery, Isfahan University of Medical Sciences, Isfahan, Iran; 3 Department of Health Education and Promotion, School of Health, Isfahan University of Medical Sciences, Isfahan, Iran; 4 Nutrition Research Center, Department of Clinical Nutrition, School of Nutrition and Food Sciences, Shiraz University of Medical Sciences, Shiraz, Iran

**Keywords:** Polycystic ovary syndrome, Nutritional behaviors, Health system, Adolescent girls, Qualitative study

## Abstract

**Background:**

Polycystic ovary syndrome (PCOS) is the most common endocrine disorder among women. Given the prevalence of this disease in adolescent girls as well as its serious physical, psychological, and social consequences, the present study aimed to explore the health system-related needs for healthy nutritional behaviors in adolescent girls with PCOS.

**Methods:**

This qualitative content analysis was conducted in Shiraz, Iran between November 2016 and October 2017. Eighteen Adolescent girls with PCOS and 15 healthcare providers (midwives, gynecologists, nutritionists, and endocrinologists) were selected through purposeful sampling with maximum variation strategy. Data were collected through individual in-depth and semi-structured interviews, focus group discussions, and field note, and simultaneously analyzed using the conventional qualitative content analysis method.

**Results:**

Three main categories that appeared included: 1) education and counseling on healthy nutrition and support for adolescent girls with PCOS with sub-categories of “empowering adolescent girls with PCOS to adopt healthy nutritional behaviors”, “providing services and education about healthy nutritional behaviors as a team”, and “the health team attention to the concerns of adolescent girls with PCOS and closely following the disease status, 2) solving communication problems with sub-categories of “proper interactions and building trust between health team members and adolescent girls” and “proper interactions between members of the health team”, and 3) developing the optimal structure for providing health services with sub-categories of “solving problems related to human resources, “promoting the position of health issues related to adolescent girls in the health system”, and “promoting policy-making in the field of nutritional health of adolescent girls”.

**Conclusions:**

Based on the results of the present study, necessary measures should be taken to educate and advise on healthy nutrition, and to support adolescent girls with PCOS. The desired structure should also be developed to provide health services to these girls. By solving communication problems and building trust between the members of the health team and these girls, one can guide them to adopt healthy nutritional behaviors.

**Supplementary Information:**

The online version contains supplementary material available at 10.1186/s12913-022-08334-2.

## Background

Polycystic ovary syndrome (PCOS) is the most common endocrine disorder among women [1]. This syndrome is a growing concern among adolescents around the world, with varying reports of its prevalence in different parts of the world. Saei Ghare Naz et al., in a systematic review and meta-analysis, showed that there were variations in the prevalence of PCOS in adolescents based on different criteria. In this regard, the prevalence of PCOS in adolescents based on the Rotterdam criteria was 11.04% (95% CI: 6.84–16.09%), based on the National Institute of Health (NIH) criteria, it was 3.39% (95% CI: 0.28–9.54%), and according to Androgen Excess and Polycystic Ovary Syndrome Society, it was 8.03% (95% CI: 6.24–10.01%) [2]. Fattah et al., in a meta-analysis, reported that the prevalence of PCOS was 5.2% (95% CI: 3-8%) among Iranian adolescent girls [3].

In this disease, the accumulation of body fat in a central form (android obesity) and the increase in the waist-to-hip ratio cause metabolic syndrome and its complications, including increased risk of type 2 diabetes [4]. Also, following an increase in insulin and stimulation of ovarian androgen production, a permanent imbalance in sex hormones [5] leads to ovulation disorders and eventually infertility [6]. According to studies, obesity and insulin resistance are among the pathophysiological principles of PCOS [7]. Over the past few years, the rate of obesity among adolescents has increased from 51-59% between 1987 and 1999 to 74% between 2000 and 2002 [8]. This is important because obesity can accelerate the clinical manifestations of PCOS in adolescent girls with this disease [9]. Given that obesity plays a major role in the pathophysiology of PCOS, and because in most studies it has also been shown that evidence-based and socio-culturally appropriate healthy lifestyle as well as nutrition is effective in weight loss in adolescent girls with PCOS [10–12], attempts to change nutritional behaviors can not only improve the various manifestations of the disease in these people, but will also have positive effects on their quality of life and future reproductive health [13].

There are about 1.7 billion adolescents aged 10 to 19 worldwide, about 90% of whom live in developing countries [14]. In Iran, about 30% of the population are aged 10-30, of whom about 16.33% are in the age group of 10 to 19 [15]. Since the achievement of the millennium development goals (MDGs) requires a careful focus on adolescence, thus, ensuring that countries pay attention to adequacy of services in the field of adolescent health is on the agenda of the World Health Organization (WHO) [16]. Also, due to the growing trend of adolescent population by 2030, international agencies such as United Nations Children's Fund (UNICEF) and WHO have given high priority to their nutritional and reproductive health [17]. In this regard, the changeable nature of nutritional behaviors suggests the need to plan and consider appropriate approaches to correcting unhealthy nutritional behaviors for controlling PCOS by the health system (the organization of people, institutions, and resources that deliver health care services to meet the health needs of target populations) [18].

It is believed that for developing effective intervention and prevention programs for adolescents, health policymakers should consider the views of this group of the population on health [19]. Finding out about adolescent girls’ perceptions and experiences about barriers to healthy nutritional behaviors will be very important in interventions to improve and enhance their nutritional pattern. In Iran, adolescent girls with PCOS have various interactions with healthcare providers (especially gynecologists) to have their disease evaluated, managed, and treated. Thus, it is necessary to discover the views and experiences of healthcare providers about the barriers to healthy nutritional behaviors by adolescent girls with PCOS in order to provide more comprehensive care to these girls. Among the scientific research approaches, the qualitative research approach is used to discover and explain the nature of phenomena, as qualitative research is a form of social research that interprets people’s experiences and the world in which they live [20]. Considering the importance of maintaining nutritional health and ultimately the reproductive health of adolescent girls with PCOS and the lack of research on health system-related needs for healthy nutritional behaviors in these girls in Iran, the present qualitative study was conducted to explore the health system-related needs for healthy nutritional behaviors in adolescent girls with PCOS.

## Method

This qualitative content analysis is part of an extensive mixed methods study (aimed to present a comprehensive interventional program for promoting eating behaviors in adolescent girls with PCOS [21]) conducted in Shiraz city in Fars province, Iran between November 2016 and October 2017.

### Settings, sample and recruitment

In this study, the participants were adolescent girls with PCOS (*n*=18) and healthcare providers (midwives, gynecologists, nutritionists, and endocrinologists) (*n*=15) Table 1. Participating adolescent girls were selected through purposeful sampling with maximum variation strategy (in terms of age, education level, occupation, marital status, and duration of disease). Inclusion criteria were as follows: i) adolescent girls with PCOS in the age group of 15-21 and with diagnosis of PCOS by a gynecologist; ii) willingness to participate in the study and informed consent of adolescents or their parents; iii) adolescent girls with PCOS who were overweight (with a body mass index (BMI) of 25 or more) or obese (with a body mass index of 30 or more) [22]; iv) no history of known major psychological disorders under medication; v) no history of chronic diseases such as diabetes, cardiovascular, and kidney disease; vi) and adolescent girls who were on a special diet prescribed by a doctor. In the present study, regarding the relationships and interactions between adolescent girls with PCOS and healthcare providers, they were selected as information-rich participants through purposive sampling. Inclusion criterion was different working experience in providing services to adolescent girls with PCOS. In this study, the participants were accessed through gynecology clinics as well as offices of gynecologists and midwives. Participants were recruited through face-to-face meetings, or their telephone numbers were obtained and they were subsequently telephoned. No one refused to participate or dropped out of this study once they were recruited to participate.

**Table 1:** Demographic characteristics of adolescent girls with PCOS and healthcare providers

Characteristic	Number
***Adolescent girls with PCOS***	18
**Education status**	
High school or below	13
Student in college	5
**Occupation**	
Student	15
Unemployed	3
**Duration of the disease (years)**	
Less than 1	4
More than 1	14
***Healthcare providers***	15
**Age (years)**	
Equal or less than 50	11
More than 50	4
**Work experience (years)**	
Equal or less than 10	3
More than 10	12

### Data collection

In this study, to enhance the richness of the obtained data, three methods were used to collect data, including individual in-depth and semi-structured interviews, focus group discussions (FGDs), and field note. The first author (LH) conducted the interviews and FGDs. She was a Ph.D. candidate in reproductive health in Isfahan University of Medical Sciences at the time of study and had 14 years of working experience in midwifery. Three other authors had a previous qualitative paper/report writing. MN and FM were Ph.D. academic members in Isfahan University of Medical Sciences and ME was a Ph.D. academic member in a university located in the study province. None of the authors had previous relationships with participants and centers. Prior to data collection, the first author wrote down initial preconceptions about the study topic based on her previous working experience and from literature review. According to the purpose of the study, the interview guide and FGDs guide were developed and assessed in one pilot interview before use in the study population. The pilot interview was also conducted to engage the first author (LH) as a qualitative researcher. All interviews and FGDs were recorded using an mp4 player. Interviews with adolescent girls began with the general question “what needs do you feel in the health care system to control and treat your disease through diet? Please explain?” Then, the participants’ open and interpretive responses guided the process. Interviews with healthcare providers began with the general question “what do adolescent girls with PCOS need in relation to the health care system to adopt healthy nutritional behaviors? Please explain?” (See Additional files 1 and 2 for copies of the topic guides). No one else was present at the interview besides the participants and the researchers. In this study, the first author (LH) documented her observations of the non-verbal behaviors of participants during the interviews (field notes). Thus, 21 individual interviews (lasting 30 to 85 minutes) were performed in the participants’ preferred locations. No repeat interviews were carried out. Since the *FGD* is a research technique employed to collect data through group interaction [23], two FGDs (one 90-minute session with 8 adolescent girls with PCOS and one 60-minute session with 4 healthcare providers) were conducted. Both group discussions were conducted in an agreed place such as gynecology clinics. At the group discussion sessions, the researcher acted as the facilitator and guide of the discussions, and another person was present to take notes. In the present study, the concept of “information power” was used to guide adequate sample size [24]. Since the official language in Iran is Persian, the specific language used for data collection was Persian. The statements of the participants were translated from Persian to English by a translator and then back translated into Persian to check for the consistency.

### Data analysis

In this study, data analysis was performed manually. Although in qualitative research use of software is helpful and supportive in data analysis, it cannot replace the researcher's thinking [25], and as such no software was used to analyze the data. Conventional qualitative content analysis method [20] was employed for data analysis. The first author (LH) transcribed the interviews verbatim and FGDs on a regular basis as each interview and FGDs was conducted. The interviews and FGDs were then read over and over again to gain a complete understanding of them. Coding was done by the first author (LH), with a subset of 10% of the transcripts coded independently by the second author (MN) using the developed coding frame. Based on the research objectives and interview guide, a code book was developed by the first author (LH) and shared with the research team. Coding discrepancies were resolved through discussion and consensus. Once codes were formed inductively, similar codes were merged and grouped together to form sub-categories. Then, by comparing the sub-categories with each other, the categories that were conceptually related to each other were placed in one category, and thus, the main categories were formed.

### Rigor and trustworthiness

In this study, to validate the data, various methods were used including in-depth interviews at different times and places as well as a combination of several data collection methods. In order to enhance transferability, the data were presented to five individuals with similar characteristics of the participants who did not participate in the study, to judge the similarity of the data with their experiences. Also, the opinions of two experts were used to match and ensure the consistency of the data with the narrations of the participants. In order to confirm the credibility of the obtained data, transcripts were returned to participants for comments or corrections. Also, in other sessions, coded interviews were shared with four participants and their final comments were summarized (member checking).

### Ethical considerations

Approval of the research was obtained from the Ethics Committee of the Vice Chancellor for Research of Isfahan University of Medical Sciences (approved code: IR.MUI.Rec.1395.8.885). In this study, anonymity, confidentiality of information, and the right to withdraw at any time were respected. Written informed consent was taken from each participant, and for the participants under the age of 18, written informed consent was received from a parent or legal guardian. Also, all participants in the focused groups provided written informed consent prior to participation. The reasons for the study were explained before each individual interview and FGD.

## Results

After analyzing the data, 163 codes, eight sub-categories, and three main categories were obtained including “education and counseling on healthy nutrition and support for adolescent girls with PCOS”, “solving communication problems”, and “developing the optimal structure for providing health services” Table 2.

**Table 2:** Results of data analysis

Codes	Sub-category	Main category
-Need for continuous encouragement of adolescent girls to follow the correct nutrition pattern by a team of different experts (gynecologist, nutritionist, endocrinologist, sport specialist, psychologist) -The need to follow the process of weight loss of adolescent girls by a team consisting of different experts -The need for continuous training in healthy nutritional behaviors by a team of different experts	Providing services and education about healthy nutritional behaviors as a team	Education and counseling on healthy nutrition and support for adolescent girls with PCOS
-Need to increase adolescent girls’ awareness of problems caused by poor eating habits -Need to increase adolescent girls’ awareness of healthy nutritional behaviors -Need to increase the awareness of adolescent girls about the role of weight loss in improving the disease -Need to increase the awareness of adolescent girls about the importance of the disease in their reproductive health - Need to increase the awareness of adolescent girls about the nature and complications of the disease	Empowering adolescent girls with PCOS to adopt healthy nutritional behaviors	
-Providing inadequate and brief explanations about the disease by various experts -Stress and anxiety of adolescent girls about the complications of the disease -Confusion of adolescent girls after seeing different doctors -Low quality of doctor’s visits and not spending enough time - Failure to justify adolescent girls about the need for careful follow-up of the disease status	The health team attention to the concerns of adolescent girls with PCOS and closely following the disease status	
-Lack or inefficiency of nutrition consultants in health centers - Lack of health educators in schools - Lack of nutrition expert in schools - Lack or shortage of trained health workers	Solving problems related to human resources	Developing the optimal structure for providing health services
-Not taking the health problems of adolescent girls in the health system seriously - Lack of centers to monitor the health of adolescent girls -Need to develop public health promotion programs for adolescent girls -Not considering the reproductive health needs of adolescent girls in macro planning	Promoting the position of health issues related to adolescent girls in the health system	
-Poor policy-making and correct and practical planning for adolescents to adopt a healthy lifestyle -Poor implementation of healthy lifestyle action plans in adolescents -Poor monitoring and evaluation system of adolescent health programs	Promoting policy-making in the field of nutritional health of adolescent girls	
-Adolescent girls’ dissatisfaction with the reactions and behaviors of some healthcare providers -Adolescent girls’ lack of trust in some health care providers - Receiving conflicting information from healthcare providers	Proper interactions and building trust between health team members and adolescent girls	Solving communication problems -Need for healthcare providers to have the necessary communication skills
-Need for interaction between gynecologists and nutritionists -Need for interaction between gynecologists and endocrinologists -Need for accountability of different experts -Need for cooperation of different experts	Proper interactions between members of the health tea	

### Education and counseling on healthy nutrition and support for adolescent girls with PCOS

According to the participants, education and counseling on healthy nutrition and support for adolescent girls with PCOS were among the health system-related needs that could affect their nutritional behaviors. This main category consisted of three sub-categories.

#### Empowering adolescent girls with PCOS to adopt healthy nutritional behaviors

The participating girls believed that their knowledge of the nature of their disease was very limited. They stated that if they knew the side effects of the disease and its connection with nutritional behaviors, they would further follow the doctors’ recommendations to lose weight. Participating healthcare providers also underestimated girls’ awareness of the disease and its effect on reproductive health. They noted the need for nutritional health education, introduction of healthy eating habits, and promotion of a healthy lifestyle culture among adolescent girls with PCOS. Healthcare providers found that making adolescent girls with PCOS aware of the role of weight loss in improving the disease was very effective in empowering them to adopt healthy nutritional behaviors and to strive to lose weight. A nutritionist stated:

*“... It is very effective when each person in their field of expertise explains healthy nutritional behaviors related to weight loss to these girls and they would be well justified about the consequences of healthy nutritional behaviors ...” (Participant No. 25)*

### Providing services and education about healthy nutritional behaviors as a team

According to the participants, one of the effective ways to adopt healthy nutritional behaviors in adolescent girls with PCOS is to provide services and education about healthy nutritional behaviors in the form of a team consisting of a nutritionist, gynecologist, endocrinologist, sports specialist, and psychologist. The participants noted the importance of continuous nutrition training by emphasizing the side effects of not following nutritional recommendations and following the weight loss process by team members. A nutritionist said:

“*One of the factors that has a great effect on weight loss and healthy nutritional behavior is establishing a team of different professionals ...” (Participant No. 27).*

### The health team attention to the concerns of adolescent girls with PCOS and closely following the disease status

The participating girls narrated that no one in the health system is willing to answer their questions and concerns about their illness properly, which aggravates their worries and stress. They expressed dissatisfaction with the low quality of the visits and the lack of sufficient time for the visit. One of the girls said:

*“… When I go to the doctor, without asking questions about my disease, she just spends her time writing a few medications and urges me to lose weight, that’s it!” (Participant No. 1).*

Participants considered healthcare providers justifying adolescent girls with PCOS about the nature and complications of the disease, the need to closely monitor the disease status, and the importance of the disease in reproductive health as essential items that can reduce their anxiety. One gynecologist said:

*“.... Unfortunately, most colleagues, since they are busy, do not spend much time and prescribe a few pills quickly, and tell the adolescent girl to go and see her again in 6 months. The patient should be explained about the status of her disease and its importance!!!” (Participant No. 30).*

### Solving communication problems

According to the participants, solving communication problems as well as appropriate interactions between health team members and adolescent girls with PCOS alongside appropriate interactions between health team members are among the needs related to the health system that can affect the nutritional behaviors of adolescent girls with PCOS. This main category consisted of two sub-categories.

### Proper interactions and building trust between health team members and adolescent girls

Many of the participating girls were dissatisfied with the reactions and behaviors of the health team members in dealing with them, believing that the discomfort and boredom prevented them from following their doctors’ recommendations for weight loss to improve and control their disease. The participating girls believed that healthcare providers lacked the necessary communication skills to deal with them and could not meet their expectations. One of the girls said:

*“... I’m really upset with the health system and its staff. ... Not only is their behavior disrespectful, but when I have a question or I am looking for an answer to my questions, they give me a vague answer.” (Participant No. 11).*

According to some adolescent girls, they encounter contradictory information and treatments when referring to different doctors, which has led to their loss of trust, making it difficult for them to recognize the disease and its complications, and confusing them in the treatment process. Many adolescent girls cited receiving encouragement and support from doctors, especially nutritionists, as a strong motivating factor for weight loss. Another girl stated:

*“… Well, I do not want much; I just want someone to listen to me! Someone who understands me, to get out of worry!” (Participant No. 16).*

### Proper interactions between members of the health team

Participating healthcare providers reported that one of the reasons for not taking doctors’ advice to lose weight seriously by adolescent girls with PCOS is the lack of proper interaction and responsibility among health team members and girls’ perception of the health team members’ inability to treat their disease. According to the participants, proper interaction between healthcare providers will fully justify adolescent girls about their disease as well as the need to lose weight and follow a special diet, thus increasing their chances of controlling and managing PCOS. A gynecologist said:

*“I usually refer PCOS patients to a nutritionist and an endocrinologist, and patients are very satisfied with this referral. ... It usually works best when patients are under the care of several specialists.” (Participant No. 29).*

One of the midwives said:

*“The important thing is that PCOS is a syndrome and the range of its symptoms and complications is very wide requiring the full cooperation of gynecologists, nutritionists, and endocrinologists in controlling as well as managing this disease.” (Participant No. 36).*

### Developing the optimal structure for providing health services

According to the participants, the development of an optimal structure for providing health services is one of the factors that can affect the nutritional behaviors of girls with PCOS. This main category includes 3 three sub-categories.

#### Solving problems related to human resources

According to the participants, the health system does not have sufficient and efficient human resources in dealing with adolescent girls and solving problems related to nutritional health, as well as guiding them to adopt healthy nutritional behaviors. Lack of trained healthcare providers, lack of or inefficient nutrition counselors in health centers, lack of health educators in schools, and absence of nutritionists in schools were all mentioned by participants. The endocrinologist stated:

*“… Unfortunately, the lack of human resources and expertise is effective in correcting the nutritional behaviors of adolescent girls and leading them to healthy nutritional behaviors. Some schools do not have health educators. We may not have an active efficient nutrition expert in clinics.” (Participant No. 39).*

### Promoting the position of health issues related to adolescent girls in the health system

According to the participants, issues and problems related to the health of adolescent girls, especially reproductive health, are not very important from the perspective of policymakers. They believed that although girls’ health is in fact a guarantee of family health, the health system’s reproductive health programs are mainly related to prenatal, delivery, and postpartum care. They stressed that there are no centers to monitor the health of adolescent girls as future mothers and nurturers of a healthy generation. A nutritionist said:

*“… In the health system, no one seriously cares about adolescent girls in practice, let alone their nutrition!!!” (Participant No. 23).*

### Promoting policy-making in the field of nutritional health of adolescent girls

The participants considered lack of proper and practical planning to adopt a healthy lifestyle in adolescents, poor implementation of operational healthy lifestyle programs, poor monitoring and evaluation system of adolescent health programs, poor cooperation in the implementation of instructions issued by the Ministry of Health about healthy lifestyle, and executive problems of healthy lifestyle guidelines in adolescents as obstacles to adopting healthy nutritional behaviors in adolescents. They believed that it was necessary to pay more attention to the nutritional health of adolescents in the health system. They considered establishing adolescent-friendly health services as the first step to achieving this. Most of the participants emphasized allocating specialized centers for adolescent health in accordance with their health needs and informing them about the existence of such centers. A participant from the adolescent, youth, and school health unit said:

*“… We should have adolescent-friendly centers in the comprehensive health plan of the country, where adolescents feel safe and trusted. With the adolescent and youth healthcare system, it is possible to manage the health promotion of this age group and develop healthy nutritional behaviors in them.” (Participant No. 35).*

## Discussion

This study aimed to explore the health system-related needs for healthy nutritional behaviors in adolescent girls with PCOS. The findings indicated that “education and counseling on healthy nutrition and support for adolescent girls with PCOS”, “solving communication problems”, and “developing the optimal structure for providing health services” are among the most important needs related to the health system in these people.

Based on the results of the present study, empowerment of adolescent girls with PCOS is required to adopt healthy nutritional behaviors and to strive to lose weight through education as well as counseling on healthy nutritional practices. Another study showed that educating adolescent girls about PCOS would help them gain the information they need to follow up and control the disease, which can help prevent the complications of PCOS [26]. According to the results of the present study, education about healthy nutritional behaviors in the form of team training is one of the effective strategies for adopting healthy nutritional behaviors in adolescent girls with PCOS. In this regard, Alipour et al. found that providing education and health services as a team plays an important role in the success of health programs in adolescent girls and makes them welcome adolescent-friendly service programs [27].

Based on the results of the present study, the health team should pay attention to the concerns of adolescent girls with PCOS and closely monitor their disease status. In this regard, not allocating enough time by physicians to visit adolescent girls with PCOS caused their dissatisfaction causing them not to take the doctor’s advice seriously. Nasiri Amiri et al. concluded that an important principle for treating and preventing the complications in adolescent girls with PCOS is to accept responsibility, to allocate appropriate time, and to address their concerns by healthcare providers [28]. In the study by Zarei et al., not allocating enough time by healthcare providers to adolescents was evaluated as one of the important components of health service quality [29].

In the present study, the need for appropriate interactions between adolescent girls with PCOS and healthcare providers was mentioned. Adolescent girls’ trust in the healthcare system, which has been noted in other studies as one of the main elements of appropriate communication between health team members and them [30], with positive effects on nutritional behaviors of adolescent girls with PCOS. It provides encouragement and greater acceptance of the services provided. It would also allow them to use the instructions and training provided in the field of dieting correctly and, with pleasant experiences, to recommend the use of services to others. Appropriate communication between health team members and girls will also provide healthcare providers with the opportunity to have a more accurate assessment of the current state of adolescent health and care services required [31]. Banaei et al.’s study showed that one of the main barriers to adolescent girls’ access to reproductive health services was the inappropriate behavior of service providers as well as poor quality services [32]. In the study of Damari et al., the appropriate treatment of adolescent girls by service providers was identified as one of the important components of the quality of health services [33].

According to the present study results, the existence of appropriate interaction among healthcare providers as well as their sense of responsibility would encourage adolescent girls with PCOS to refer to specialists and follow a proper diet to control their disease. In the review study of Brittain et al., the lack of proper interaction between service providers and improper management of services provided were mentioned as the most important barriers to adolescents’ access to reproductive health services [31]. Also, in the study of Mulaudzi et al., inappropriate interaction between service providers and their irresponsibility was mentioned as one of the most important reasons for adolescent girls not using the health services they need [19].

According to the results of the present study, one of the needs related to the health system in the field of promoting nutritional behaviors among adolescent girls with PCOS is to solve problems related to human resources and the presence of trained staff. In this regard, it is necessary to employ health workers who have appropriate scientific and functional skills in dealing with adolescents. The results of a study revealed that the skills of healthcare providers would provide a friendlier environment for adolescents, which in turn would lead to more acceptance of the services provided [34]. A study in Jordan, aiming at explaining adolescents’ views on reproductive health services, also indicated that the staff of reproductive health centers did not have sufficient capabilities and skills to communicate properly with adolescents [35]. It is believed that although achieving global standards in the field of performance of healthcare providers may require the creation of some new organizational positions, training existing staff is one of the most cost-effective tasks in the short term [36].

Based on the results of the present study, improving the status of adolescent girls’ health issues in the health system and promoting policy-making in the field of adolescent girls’ nutrition are other needs related to the health system in promoting nutritional behaviors in adolescent girls with PCOS. According to the participants, the needs of adolescent girls in the field of health issues are not properly addressed and issues related to adolescent health have no place in the health system, policymaking, and planning. It seems that the health system in the field of adolescent health management has not yet been developed. Thus, the following are important: planning and policy-making, financing, and management based on expert opinions regarding the conditions and problems related to the nutritional health of adolescent girls with PCOS, as well as setting specific indicators and goals for adolescent health promotion programs. According to the results of the present study, it is necessary to pay attention to the adolescents’ nutritional health in Iran’s health system, for which establishing adolescent-friendly health services was proposed. In this regard, in the study of Rahmanian et al., the need to establish and access to adolescent-friendly health services was noted [37]. The structure of providing health services related to adolescent girls can start from integrating the services they need in the primary healthcare program and continuing with the quantitative as well as qualitative development of specialized services to adolescents based on their specific needs and focusing on adolescent-friendly health services. In this regard, state centers seem to be the most appropriate option to provide the health services required by this age group, across the entire country. In this case, meeting the needs of adolescent girls with PCOS, especially regarding nutritional health, is a complex task that is beyond the reach of one institution or a group alone and requires the support and synchronization of different institutions. Thus, attracting the support of parents, schools, and religious leaders as well as their interaction with health officials and adolescents together with the support of the mass media, can be of great help in this regard [38]. In addition, attracting the participation of charities and NGOs in promoting adolescent health services is among the policies recommended and used in some countries with a similar cultural context to Iran [39].

### Strengths and limitations

Through presenting an image of health system-related needs for healthy nutritional behaviors in adolescent girls with PCOS, for the first time in Isfahan city, Iran, the present study could help in designing the necessary interventions to improve nutritional behaviors in these people. Also, the results of this study can be the basis for conducting wider and more comprehensive studies about nutritional behaviors in adolescent girls with PCOS.

The findings of the present qualitative research provide an understanding of the health system-related needs for healthy nutritional behaviors in adolescent girls with PCOS. However, due to qualitative nature of this research, its generalizability to other social groups may be limited and is considered one of its limitations. Also, although the results of the present qualitative research can provide a deep understanding of health system-related needs for healthy nutritional behaviors in overweight and obese adolescent girls with PCOS, the results may not be shared by normal weight adolescent girls with PCOS. Since nutritional behaviors in normal weight adolescent girls with PCOS can be different, future studies are recommended to explore health system-related needs for healthy nutritional behaviors in normal weight adolescent girls with PCOS.

## Conclusions

The results of the present study, by explaining and highlighting the health-related needs of adolescent girls with PCOS, could be used to make policies and design successful interventions as well as to provide principled and comprehensive care programs for adopting healthy nutritional behaviors. In this regard, necessary measures should be taken to educate and advise on healthy nutrition, and to support adolescent girls with PCOS. The desired structure should also be developed to provide health services to these girls. By solving communication problems and building trust between the members of the health team and these girls, it would be possible to guide them to adopt healthy nutritional behaviors.

## Supplementary Information

**Additional file 1.** Interview and FGDs guides during the face-to-face interviews and FGDs for the study conducted to explore health system-related needs for healthy nutritional behaviors in adolescent girls with PCOS from the perspective of adolescent girls with PCOS in Shiraz Town, Iran, 2016 - 2017.

**Additional file 2.** Interview and FGDs guides during the face-to-face interviews and FGDs for the study conducted to explore health system-related needs for healthy nutritional behaviors in adolescent girls with PCOS from the perspective of healthcare providers (midwives, gynecologists, nutritionists, and endocrinologists) in Shiraz Town, Iran, 2016-2017.

## Data Availability

The datasets generated and/or analyzed during the current research are not publicly available as individual privacy could be compromised but are available from the corresponding author on reasonable request.
