# Supplementary material for: Health system-related needs for healthy nutritional behaviors in adolescent girls with polycystic ovary syndrome (PCOS): a qualitative study in Iran
Source: BMC Health Serv Res. 2022 Aug 5;22:998. doi: 10.1186/s12913-022-08334-2 (PMC9354299; doi:10.1186/s12913-022-08334-2)
Supplement: Supplementary file 1 — Additional file 1. Interview and FGDs guides during the face-to-face interviews and FGDs for the study conducted to explore health system-related needs for healthy nutritional behaviors in adolescent girls with PCOS from the perspective of adolescent girls with PCOS in Shiraz Town, Iran, 2016 - 2017. [file 12913_2022_8334_MOESM1_ESM.docx]

**Additional file 1:** Interview guide and FGDs guide during the face-to-face interviews and FGDs for the study conducted to explore health system-related needs for healthy nutritional behaviors in adolescent girls with PCOS from the perspective of adolescent girls with PCOS in Shiraz Town, Iran, 2016 - 2017.

(See methods section for further description).

**Introduction:** *Aim, to create appropriate atmosphere*

- Name of the interviewer and affiliation
- Purpose of the study
- Consent to take part in the study
- Confidentiality, explain how the data will be used
- Audio recorded to ensure interviewer can fully engage in the interview

**Warm up questions:** *Aim\ make participants comfortable*

1. Please introduce yourself?

2. How old are you?

3. What is your education level?

4. What is your job?

**Interview guide questions in individual interviews and FGDs with adolescent girls with PCOS**

1.What needs do you feel in the healthcare system to control and treat your disease through diet? Please explain?

2. How can healthcare providers (midwives, gynecologists, nutritionists, and endocrinologists) help you? Please explain?

3. What would you like healthcare providers to do for you? Please explain?

4. In general, what do you expect from the health system to improve your situation?
