# Supplementary material for: Health system-related needs for healthy nutritional behaviors in adolescent girls with polycystic ovary syndrome (PCOS): a qualitative study in Iran
Source: BMC Health Serv Res. 2022 Aug 5;22:998. doi: 10.1186/s12913-022-08334-2 (PMC9354299; doi:10.1186/s12913-022-08334-2)
Supplement: Supplementary file 2 — Additional file 2. Interview and FGDs guides during the face-to-face interviews and FGDs for the study conducted to explore health system-related needs for healthy nutritional behaviors in adolescent girls with PCOS from the perspective of healthcare providers (midwives, gynecologists, nutritionists, and endocrinologists) in Shiraz Town, Iran, 2016-2017. [file 12913_2022_8334_MOESM2_ESM.docx]

**Additional file 2:** Interview guide and FGDs guide during the face-to-face interviews and FGDs for the study conducted to explore health system-related needs for healthy nutritional behaviors in adolescent girls with PCOS from the perspective of healthcare providers (midwives, gynecologists, nutritionists, and endocrinologists) in Shiraz Town, Iran, 2016-2017 (See methods section for further description).

**Introduction:** *Aim, to create appropriate atmosphere*

- Name of the interviewer and affiliation
- Purpose of the study
- Consent to take part in the study
- Confidentiality, explain how the data will be used
- Audio recorded to ensure interviewer can fully engage in the interview

**Warm up questions:** *Aim\ make participants comfortable*

1. Please introduce yourself?

2. How old are you?

3. What is your education level?

4. What is your job?

5. What is your work experience?

**Interview guide questions in individual interviews and FGDs with healthcare providers**

1. In your opinion, what do adolescent girls with PCOS need in relation to the healthcare system to adopt healthy nutritional behaviors? Please explain?

2. What can healthcare providers do to meet the needs of these girls?

3. In your opinion, what should health policymakers do to improve the status of adolescent girls with PCOS?
